# Comparison of the Gene Expression Profiles of Human Fetal Cortical Astrocytes with Pluripotent Stem Cell Derived Neural Stem Cells Identifies Human Astrocyte Markers and Signaling Pathways and Transcription Factors Active in Human Astrocytes

**DOI:** 10.1371/journal.pone.0096139

**Published:** 2014-05-21

**Authors:** Nasir Malik, Xiantao Wang, Sonia Shah, Anastasia G. Efthymiou, Bin Yan, Sabrina Heman-Ackah, Ming Zhan, Mahendra Rao

**Affiliations:** 1 National Institutes of Health, NIAMS, Bethesda, Maryland, United States of America; 2 Hong Kong Baptist University, Department of Biology, Hong Kong; 3 The Methodist Hospital Research Institute, Weill Cornell Medical College, Houston, Texas, United States of America; 4 National Institutes of Health, NIH Center for Regenerative Medicine, Bethesda, Maryland, United States of America; University of Melbourne, Australia

## Abstract

Astrocytes are the most abundant cell type in the central nervous system (CNS) and have a multitude of functions that include maintenance of CNS homeostasis, trophic support of neurons, detoxification, and immune surveillance. It has only recently been appreciated that astrocyte dysfunction is a primary cause of many neurological disorders. Despite their importance in disease very little is known about global gene expression for human astrocytes. We have performed a microarray expression analysis of human fetal astrocytes to identify genes and signaling pathways that are important for astrocyte development and maintenance. Our analysis confirmed that the fetal astrocytes express high levels of the core astrocyte marker GFAP and the transcription factors from the NFI family which have been shown to play important roles in astrocyte development. A group of novel markers were identified that distinguish fetal astrocytes from pluripotent stem cell-derived neural stem cells (NSCs) and NSC-derived neurons. As in murine astrocytes, the Notch signaling pathway appears to be particularly important for cell fate decisions between the astrocyte and neuronal lineages in human astrocytes. These findings unveil the repertoire of genes expressed in human astrocytes and serve as a basis for further studies to better understand astrocyte biology, especially as it relates to disease.

## Introduction

Astrocytes are process-bearing glial cells that comprise at least 20–25% and possibly up to 50% of the total volume in some regions of the central nervous system (CNS). They play an integral role in normal homeostasis of the adult brain providing trophic support to neurons and oligodendrocytes as well as degrading potential toxins. Amongst many other functions, astrocytes have important roles in glutamate biology, axonal guidance, the inflammatory response and wound healing, formation of the blood brain barrier, iron metabolism, and myelination [1]. Because astrocytes have such diverse functions it is not surprising that they have been implicated in many human diseases including amyotrophic lateral sclerosis, epilepsy, and Parkinson's disease [2].

A great deal of progress has been made in understanding astrocyte development in recent years. Like all other neural cells in the CNS, astrocytes develop from multipotent neuroepithelial cells or neural stem cells (NSCs). Neuronal formation precedes gliogenesis in the course of normal development. Studies of astrocyte development have revealed that the bone morphogenetic protein (BMP), fibroblast growth factor-2 (FGF2), signal transducer and activators of transcription (STAT), heregulin, and NOTCH signaling pathways are critical for proper formation of this cell type with considerable cross-talk between these pathways [3]–[7]. BMPs activate basic helix-loop-helix (bHLH) factors from the ID and HES families to repress proneural bHLH factors and initiate gliogenesis [8]. It is believed that this differentiation program is locked into place by induction of the neuronal restrictive silencing factor (NRSF) which inhibits neurogenesis and promotes gliogenesis [9]. Studies also suggest that the transcription factor NFIA is critical for the initiation of gliogenesis by inducing expression of the Notch effector HES5 [10]. NFIA expression in turn is regulated by SOX9 which complexes with NFIA to turn on a gliogenesis transcriptional program [11]. When overexpressed in glioma cells, NFIX, a paralog of NFIA, has also been shown to regulate astrocyte maturation by activating expression of several genes found in mature astrocytes [12]. Mouse knock-out studies have not been completely conclusive about the role of NFI and SOX genes in gliogenesis because of redundancy in these gene families but they do suggest that SOX9 and NFIA have a role in glial, particularly astroglial, development [13]–[14]. Another pathway that has been shown to have a critical role is gliogenesis is the MAPK pathway which can regulate differentiation of astrocytes and oligodendrocytes through MEK activation [15]. It is likely that there is additional crosstalk between the transcription factors and signaling pathways described above that has yet to be elucidated.

Cell culture models in which NSCs are differentiated to astrocytes and subsequent global gene expression analysis of a homogeneous population of cells can help identify new genes that are pivotal for astrogenesis. These types of analyses can also offer insights into the transcriptome of mature astrocytes and will be particularly useful for studying human astrocytes. Previously microarray data has only been available for murine astrocytes and human glial precursors [16]–[17]. We have recently published a paper describing a new method for the differentiation of astrocytes from human pluripotent stem cells (PSCs) using heregulin-β1 and performed a time-course microarray analysis from days 14–35 of astrocyte differentiation [18]. In this current study we have expanded the array datasets to include comparisons of human fetal astrocytes from two commercial suppliers comparing our results with those of the previous studies to identify genes and signaling pathways that are critical for astrocyte maturation and function. The availability of this data will make it possible to test and verify predictions about the importance of specific pathways and genes in astrocyte development and biology. Additionally, the data offers a new series of potential unique astrocyte markers that can be used to ascertain the purity of astrocyte populations that are differentiated from NSCs. Such information will be particularly useful for the large scale production of homogeneous astrocyte cultures for high throughput drug screens and for understanding neurological disorders resulting from astrocyte dysfunction.

## Materials and Methods

### Cell culture

Astrocytes from human fetal cortex were purchased from Lonza (Walkersville, MD USA) and ScienCell (Carlsbad, CA USA) and grown according to manufacturer's instructions. The Lonza astrocytes were grown on uncoated tissue culture plates in Lonza's Astrocyte basal medium with L-glutamine, gentamicin, 3% FBS and supplemented with Lonza's astrocyte growth medium SingleQuots (ascorbic acid, EGF, insulin). The cells were passaged with trypsin/EDTA once they reached 80% confluence. ScienCell astrocytes were grown on poly-L-lysine coated plates in the company's astrocyte medium and passaged with trypsin/EDTA once the plates were 80% confluent.

NSCs used in this study were either derived from human PSCs using an embryoid body (EB) intermediate as previously described [19], derived by direct induction [20], or purchased from Life Technologies and cultured in StemPro NSC SFM medium ((Life Technologies, Grand Island, NY, USA). The direct induction protocol utilizes a Neural Induction medium (Life Technologies) in which PSCs are initially seeded at a low density and grown for one week to become neuroepithelial cells, then switched over to a neural expansion medium (Life Technologies) for generation of NSCs that can be passaged and cryopreserved. Once the NSC lines were established they were grown in StemPro NSC SFM consisting of KO-DMEM/F12, StemPro Neural Supplements, 20 ng/ml FGF2, and 20 ng/ml EGF (all from Life Technologies). The differentiated neurons used in this study were derived from iPSC-derived NSCs with neuronal differentiation medium as previously described [21] and iPSC-derived astrocytes used in this study were generated with a slight modification of previously described astrocyte differentiation medium [18] consisting of DMEM/F12, GlutaMAX-I, B27 supplement, 8 ng/ml FGF2, and 10 ng/ml of heregulin-β1 (all components from Life Technologies except heregulin which was from Peprotech (Rocky Hill, NJ USA).

### Immunofluorescence

Cells were processed for staining by fixation in 4% paraformaldehyde for 10 minutes at room temperature followed by three washes in PBS. They were then incubated in blocking buffer containing 10% goat serum, 0.1% Triton-X, and 1% bovine serum albumin for 30 minutes followed by overnight incubation at 4°C with primary antibodies in blocking buffer. Primary antibodies used in this are described in Table S1. After removal of primary antibody the cells were washed with PBS three times and incubated with Alexa fluor secondary antibodies and Hoechst in blocking buffer (1∶500 for Alexa and 1∶2000 for Hoechst, both from Life Technologies) for two hours at room temperature. Staining was visualized on a fluorescence microscope with the appropriate filter settings.

### Bead array hybridization

The Lonza astrocytes were grown in a T75 to 80% confluence and cells collected for microarray hybridization after the first passage. Astrocytes from ScienCell were grown in a 6 cm cell culture dish, passaged once into a T75, grown to 80% confluence and collected for microarray hybridization. Pelleted cells were sent to Qiagen (Frederick, MD USA) for extraction, amplification, labeling and hybridization of RNA to an Illumina HT-12 v4 BeadChip array. The.idat files for the arrays were sent to us for analysis.

### Bead array analysis

The Gene Expression module of the Illumina GenomeStudio software package was used to process the.idat files. The data were normalized with background subtraction and scatter plots and dendrograms were generated. The data were exported to Excel for further “cleaning” by removing any probes in which the intensity value was <50 for all samples and all intensity values less than 1 were converted to 1. Expression fold changes for each commercial astrocyte sample compared to an NSC sample were calculated and only those genes which showed greater than 5-fold changes in expression were considered as cell-type specific markers. Gene lists from relevant developmental pathways, all human transcription factors, and growth factors and their receptors were interrogated against this dataset. This dataset was also analyzed for expression of genes found to be enriched in other astrocyte gene expression studies and from an array dataset generated from neurons differentiated from iPSC-derived NSCs [16], [18], [21].

### Transcription factor binding site analysis

We employed PWMSCAN to predict binding sites of TFs [22] This method performs computational identification of the binding sites by scanning promoter sequences using PWMs (Position Weight Matrices) of TF binding motifs. The predicted binding site is then evaluated by the *P* value, which is calculated through a permutation-based method, FastPval [23]. A sequence hit with *P-*value less than the user specified cutoff is considered as a putative binding site. Moreover, the putative binding sites can be further filtered based on conservation scores between human and mouse genomes, which are evaluated by another permutation-based test. In this study, we scanned three promoter regions: 1) −500 bp (upstream to TSS) ∼ +100 bp (downstream to TSS); 2) −2000 bp ∼ −500 bp; 3) −8000 bp ∼ +2000 bp, which were downloaded from the UCSC genome browser. We retrieved PWMs of TFs available from two databases TRANSFAC and Jaspar.

### Inhibition of NOTCH signaling with DAPT

For Notch pathway inhibition ∼100,000 NSCs were plated onto poly-L-ornithine/laminin-coated 24-well plates. The next day cells were either grown in NSC medium or the medium was changed to either neuronal differentiation medium or astrocyte differentiation medium. The Notch pathway inhibitor DAPT (Sigma Aldrich, St. Louis, MO USA) was added to the medium at a concentration of 10 μM. The cells were grown for an additional four days with medium changes every other day and then processed for immunofluorescence for the neuronal marker beta-III-tubulin and the astrocyte marker GFAP. Beta-III-tubulin positive cells were quantified by calculating the number of positive cells in three separate fields of view. A t-test was performed to assess statistical significance between samples.

### Lentiviral infection

cDNAs for NFIX and HOPX in second generation lentiviral vectors were purchased form Thermo Scientific (Pittsburgh, PA USA). A control GFP lentiviral cDNA was used as well. One million NSCs plated in three wells of a 6-well plate were infected at an MOI of 10 and three days later cells were either maintained in NSC medium or switched to astrocyte or neuronal differentiation medium. Two days after the medium change the cells were analyzed qPCR for NSC, astrocyte, and neuronal markers with beta-actin as a normalization control. Lonza astrocytes were similarly infected and analyzed by qPCR three days after infection. Total RNA from GFP-control, HOPX-, and NFIX-lentivirus infected cells was extracted using RNeasy Mini Kit (Qiagen, CA). cDNA was generated from 1 µg total RNA using SuperScript III First -Strand Synthesis System kit (Life Technologies, CA). The β-actin gene was employed as an endogenous control to normalize input cDNA. qPCR reactions was performed on the Applied Biosystems ViiA 7 Real-Time PCR Sytsem (Applied Biosystems, CA) using *Fast SYBR Green Master Mix* Kit (Life Technologies, CA). The comparative *C_T_* method was used to determine the relative target mRNA quantity in samples. The primers used are described in Table S2.

## Results

### Characterizing fetal astrocyte and iPSC-derived NSCs samples used for microarray analysis

**Figure 1:**
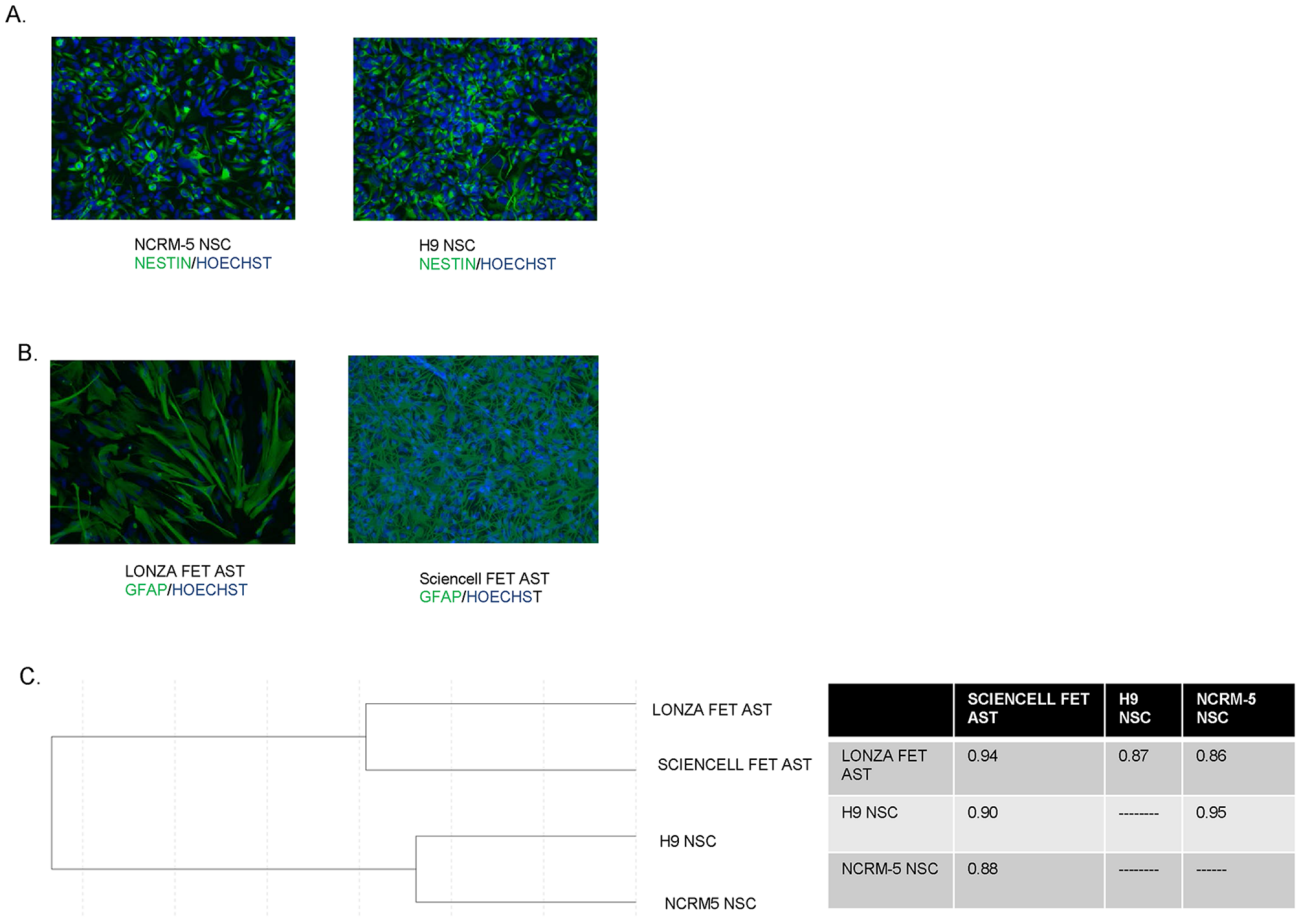
Characterization of NSC and fetal astrocyte samples. (A) NCRM-5 iPSC derived NSCs and H9 ESC derived NSCs were examined for Nestin expression (green) which was overplayed with Hoechst nuclear stain (blue) to show all cells, (B) Lonza fet ast (fetal astrocytes) and Sciencell fet ast (fetal astrocytes) were examined for GFAP expression (green) which was overplayed with Hoechst nuclear stain (blue) to show all cells, (C) dendrogram showing relationship between NSCs and fetal astrocytes at the global gene expression level and a table showing correlation coefficients amongst all samples.

In an effort to identify genes involved in regulating astrocyte development we examined the expression profile of fetal astrocytes obtained from Lonza and ScienCell and compared their expression profile to NSC lines derived from both an iPSC line by direct induction (hereafter referred to as NCRM-5 NSCs) and NSCs purchased from Life Technologies that were derived via an embryoid body (EB) based rosette method from the human H9 embryonic stem cell line (H9 NSCs). Gene expression data from previous microarray experiments indicated that NSC lines derived by either the EB/rosette or direct induction method have very similar gene expression profiles [20]. Prior to microarray hybridization the NSCs were examined for expression of the NSC marker Nestin and nearly all PSC-derived NSCs were Nestin+ (Figure 1A). Fetal astrocytes expressed GFAP, a characteristic marker of astrocytes, and showed clear differences in morphology from one another (Figure 1B) which likely reflects their distinct cell culture propagation, passage number, and isolation. Nevertheless, these cells did not show expression of NSC markers such as Sox1, and there was no neuronal contamination as assessed by β-III tubulin staining (data not shown).

The NSCs and fetal astrocytes were hybridized to an Illumina bead-chip array. Overall ∼14,000 genes were expressed above an intensity threshold of 50 in at least one of the three samples. This threshold was chosen based on our previous experience which suggested that genes expressed at intensity levels below 50 were too variable to consider in an analysis. The dataset has been deposited an is publicly available (GSE53404). Prior to gene expression analysis, dendrograms were generated to compare each of the respective fetal astrocyte samples to NSCs and to each other (Figure 1B). Despite differences in cell culture propagation both fetal astrocytes samples had a high degree of similarity to each other at the global gene expression level (R^2^ = 0.94) and formed a cluster distinct from NSCs (Figure 1C). Likewise, the two NSC lines had high correlation coefficients (0.95) whereas the fetal astrocyte and NSC samples were more dissimilar (0.86–0.90).

### Identification of fetal astrocyte-marker genes

We reasoned that if a gene was highly upregulated (at least 5-fold) in two astrocyte samples grown under different conditions compared to both of the NSC samples it would likely be a reliable candidate as an astrocyte marker. Therefore, we used this cut-off to identify new markers that are unique to this cell type. A total of 350 genes were expressed at least 5-fold higher in both fetal astrocyte samples in pairwise comparisons to the two NSC samples (Table S3). Astrocytes play important roles in immune regulation, wound healing, and modulation of the brain's vascular system and a DAVID Gene Ontology search [24] indicates that genes in these categories are amongst those most overrepresented of the genes that are at least 5-fold upregulated in fetal astrocytes (Table 1). The DAVID search also revealed that the MAPK pathway may play a critical role in fetal astrocytes which is in line with recent evidence that this pathway is important for gliogenesis to proceed [15].

**Table 1:** DAVID GO term analysis of genes enriched 5-fold in Lonza and ScienCell fetal astrocytes.

TERM	p-value	# GENES
Antigen processing	2.70E-08	8
Extracellular matrix	9.90E-07	22
Blood vessel development	1.50E-06	19
Cell adhesion	2.20E-05	17
Wound healing	8.50E-05	14
Secreted	1.30E-04	49
Inflammation	6.50E-04	8
Cell motion	1.10E-03	21
Apoptosis regulation	2.10E-03	29
MAPK activity	3.90E-03	8

In a further attempt to find genes that are most likely to be astrocyte-specific we tried to eliminate the effects of variables inherent in using primary samples by comparing the list against other datasets that we have generated. These datasets were from additional NSC lines [20], three astrocyte line differentiated from iPSC-derived NSCs (one from this study and two described in ref. 18) as well as cortical neurons differentiated from iPSC-derived NSCs (described ref 21). The array expression data for NSCs, NSC-derived neurons and astrocytes generated in this study has been publicly deposited (GSE55379). The 350 genes that were 5-fold overexpressed in fetal astrocytes were cross-checked for being expressed in the three NSC-derived astrocyte lines and either being absent from or expressed at low abundance in the other two NSC lines and in differentiated neurons. A total of 24 genes met these stringent criteria (Table 2). The list of genes includes the classical astrocyte marker GFAP and the transcription factor NFIX which is a paralog of NFIA which is known to be critical for gliogenesis [10]. Other genes of interest in this group include the transcriptional regulators LMO2, LHX2, HOPX, and PRRX1 as well as genes such as BEND6, CDKN2B, IGFBP7, LGALS3, and MVP that have known or predicted roles in signaling pathways, including NOTCH and TGF-beta, that are known to be important in astrocyte function [25]–[29].

**Table 2:** Gene highly enriched in both fetal astrocyte samples and expressed at very low levels in NSCs or differentiated neurons.

SYMBOL	LONZA FET AST	SCIENCELL FET AST	NCRM-5 NSC	H9 NSC
BEND6	216	308	3	10
CD44	3920	4352	145	233
CDKN2B	1141	440	13	17
COBL	100	399	17	7
CRYAB	1305	1230	7	1
DUSP23	1004	432	11	11
GFAP	3280	1456	8	1
HEY1	727	805	114	29
HOPX	1309	1417	210	1
IGFBP7	1487	1623	31	18
ITGA3	1056	1039	88	88
LGALS3	3055	1599	52	2
LHX2	4177	4542	2	16
LMO2	360	1674	6	1
LPPR4	431	537	1	1
MVP	266	323	16	6
NFIX	4122	1730	4	1
PRRX1	286	828	1	4
S100A6	3500	2917	40	28
SLC25A18	623	303	4	5
SYNC1	1084	395	32	8
TDRD7	264	292	5	8
TGFB3	959	156	15	10
TM4SF1	643	589	20	105

**Figure 2:**
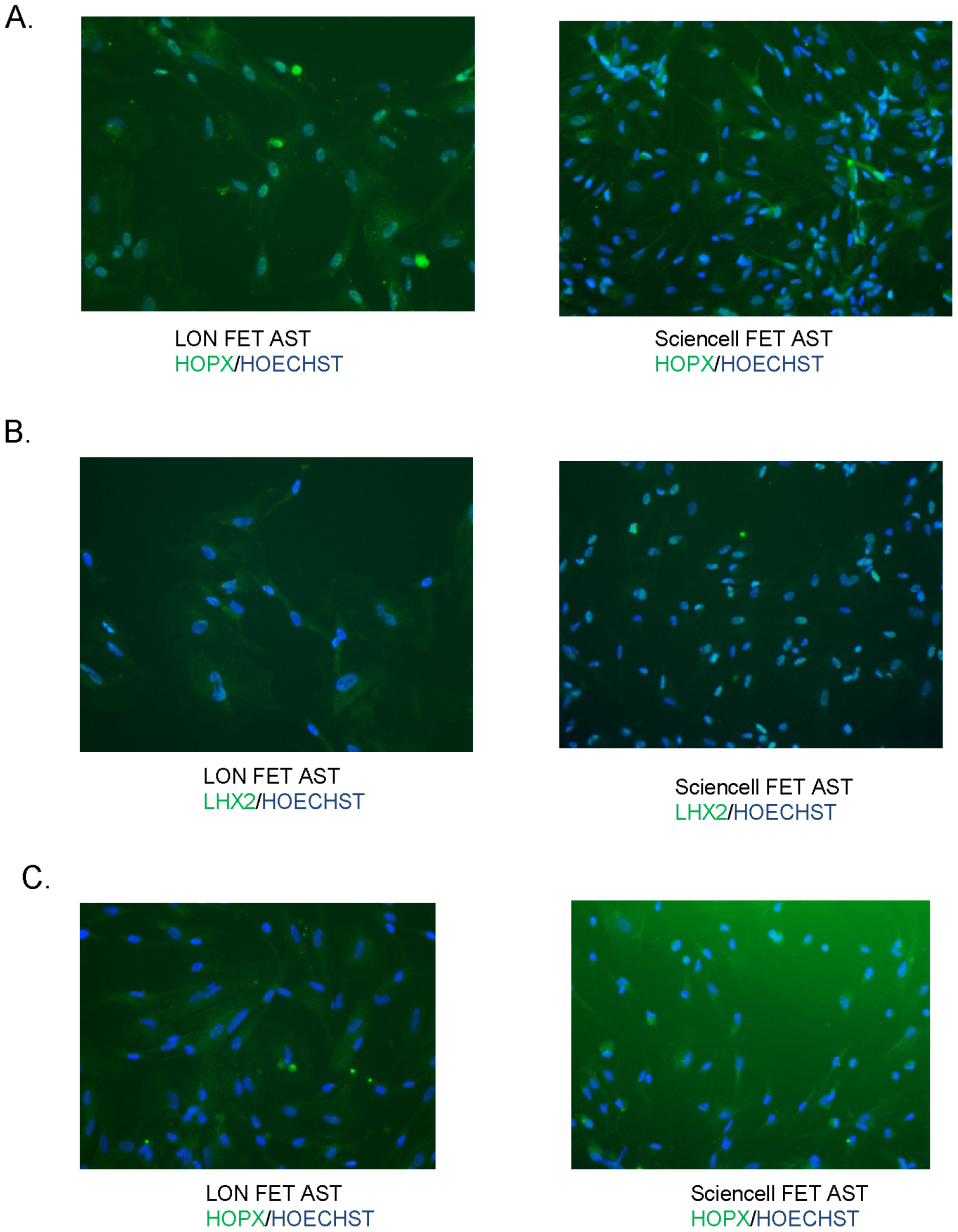
HOPX, LHX2, and PRRX1 staining of fetal astrocytes. Lonza and Sciencell fet ast (fetal astrocytes) were stained with HOPX (A), LHX2 (B), and PRRX1 (C) antibodies. The antibody signal is in green and is overplayed with HOECHST (blue) to show all cells.

We selected three transcription factors from this list to validate expression at the protein level in fetal astrocytes. LHX2, HOPX, and PRRX1 were found to be present in both fetal astrocyte lines (Figure 2A–C) and absent in NSCs by immunofluorescence (data not shown). This suggests that the genes are excellent novel marker candidates for astrocyte.

### Analysis of pathways critical for astrocyte development and maintenance

Transcription factors and signaling pathways such as NOTCH, TGF-beta, MAP kinase, and growth factor mediated pathways have been shown to play important roles in astrocyte biology. We examined each of these pathways in greater depth in our fetal astrocyte array data. Table 3 summarizes number of genes expressed and differential expression (2-fold difference) of genes amongst these pathways in fetal astrocytes and NSCs.

**Table 3:** Summary pathways analyzed in microarray.

Pathway	# Genes	Expressed 2/2 fet ast	Expressed 2/2 NSC	Up 2/2 fet ast	Down 2/2 fet ast
Transcription factors	1900	802	799	46	51
Notch	95	54	45	9	1
TGF-beta	135	54	48	5	3
JAK/STAT	206	55	49	6	4
MAPK	536	224	220	20	15
Growth factors	377	88	64	18	3
Growth factor receptors	63	15	18	3	3

#### Transcription factors

In order to generate a list of transcription factors (TFs) expressed in fetal astrocytes we interrogated our datasets against a list of all human TFs (Table S4). Table 4 displays the 46 transcription factors that are upregulated in both fetal astrocyte samples in pairwise comparisons to both NSC samples. Transcription factors in this list include the previously mentioned HOPX, PRRX1, and NFIX. Other TFs that appear to be enriched in both fetal and NSC-derived astrocytes include BHLHB2, the NOTCH effector HEY1 and the NFIX paralogs NFIA and NFIB. The remaining transcription factors appear to be more specific to fetal astrocytes. Specifically interesting ones include EMX2, FOXG1, and OTX1 all of which are known to have region specific expression in the rostral forebrain [30], [31]. Curiously the fourth member of the NFI family NFIC is enriched in fetal astrocytes but not expressed in NSC-derived astrocyte or the NSC-derived neuronal sample (this study and [18]).

**Table 4:** Transcription factors upregulated in both sets of fetal astrocytes compared to both NSC samples.

SYMBOL	LONZA FET AST	SCIENCELL FET AST	NCRM-5 NSC	H9 NSC
BCL6	280	406	54	24
BHLHB2	1233	1069	114	1
BHLHB3	145	187	2	1
CSDC2	425	189	33	21
CXXC5	3189	1658	625	715
DPF3	267	149	11	1
EGR1	3970	3779	1197	443
EMX2	763	311	11	4
ETS2	207	114	23	24
FOS	606	3343	198	63
FOXG1	547	1021	1	14
GLIPR1	850	854	4	64
GLIS3	162	157	68	48
GTF2F2	4033	3423	1437	1564
HEY1	727	805	114	29
HOPX	1309	1417	210	1
ID1	708	389	166	8
IRF9	1394	735	260	168
JUN	2010	1435	605	672
KLF5	165	275	14	7
KLF6	874	879	302	406
KLF9	724	616	132	2
LHX2	4177	4542	2	16
MBNL1	370	504	114	159
NFE2L3	705	200	44	93
NFIA	335	343	80	1
NFIB	5565	3405	1012	287
NFIC	242	175	9	7
NFIX	4122	1730	4	1
NR2E1	529	776	13	8
OTX1	183	100	1	10
PRDM16	161	113	8	3
PRRX1	286	828	1	4
RBM20	133	320	62	12
RUNX2	157	169	29	67
ZBTB20	450	943	72	81
ZBTB4	824	818	303	275
ZC3HAV1	312	291	135	121
ZFP3	202	212	19	9
ZFP36	218	333	69	102
ZNF135	368	216	41	38
ZNF385D	387	101	39	2
PHF11	638	512	9	2
PHF15	152	199	18	7
SETD6	196	129	16	23
SMARCA2	897	1052	238	188

Table 5 lists the 52 transcription factors downregulated in fetal astrocytes compared to NSCs. Genes in this list included the previously identified NSC marker MYCN, SALL4, LIN28, and LIN28B which are all well-known markers for other stem cell populations [32]–[34].

**Table 5:** Transcription factors upregulated in both sets of NSC samples compared to both fetal astrocytes.

SYMBOL	LONZA FET AST	SCIENCELL FET AST	NCRM-5 NSC	H9 NSC
ARID3B	72	33	431	737
CBX2	155	80	695	494
CHD7	277	388	2277	1863
CHX10	1	16	204	108
CXXC4	51	51	827	287
CXXC6	17	26	200	220
E2F2	210	301	1064	834
EBF1	19	1	146	146
ETV4	36	54	217	143
EZH2	204	253	740	533
FLI1	32	13	177	501
GLI2	71	48	189	176
H1FX	268	297	629	606
HES6	551	527	3211	1653
HIC2	153	77	800	1039
IRX2	70	23	410	2254
IRX5	48	29	458	342
KNTC1	372	473	1495	1122
LIN28	1	1	276	1218
LIN28B	1	6	649	1955
MYCN	74	90	1299	785
MYST3	643	621	1624	1489
PBX2	104	144	327	299
PHF16	106	101	350	332
PKNOX2	141	102	751	304
PLAGL2	182	237	536	532
POU3F2	839	944	3439	2596
PRDM8	22	36	2559	108
RAPGEF5	22	17	146	108
RCOR2	57	43	1187	663
SALL2	597	541	2225	1498
SALL4	39	17	458	1024
SORBS2	176	177	1337	2257
SOX3	351	75	3147	1629
STAT5B	75	44	151	172
SUV420H1	140	111	307	285
TCF7L1	13	17	184	104
TRIT1	382	380	879	857
ZBTB34	53	47	174	175
ZBTB39	41	22	116	85
ZBTB46	56	3	263	350
ZFHX3	181	212	479	745
ZFP14	26	5	108	52
ZMYND8	114	114	415	361
ZNF138	33	34	114	96
ZNF219	157	193	987	761
ZNF431	47	30	108	95
ZNF443	30	44	138	105
ZNF462	429	402	1759	1220
ZNF679	450	425	1167	1551
ZNF696	115	83	256	266

A subset of transcriptional regulators that was examined was genes involved with chromatin dynamics (Table S5). Six chromatin binding genes were upregulated and nine downregulated in both sets of fetal astrocytes. Of the genes upregulated in fetal astrocytes SMARCA2 and SETD6 were similarly regulated in NSC-derived astrocytes. SMARCA2 but not SETD6 was upregulated in NSC-derived neurons relative to NSCs. Several members of this gene group were also downregulated in both fetal and NSC-derived astrocytes including ARID3A, ARID3B, and EZH2. Each of these genes was also downregulated in NSC-derived neurons.

#### Signaling pathway analysis of microarray data

Four signaling pathways that are critical for astrocyte development are NOTCH, TGF-beta, JAK-STAT, and the MAP kinase pathways. We analyzed each of these pathways in our array dataset (Table S6–S9). We also examined growth factors and growth factor receptor expression (Table S10).

#### NOTCH Pathway

**Figure 3:**
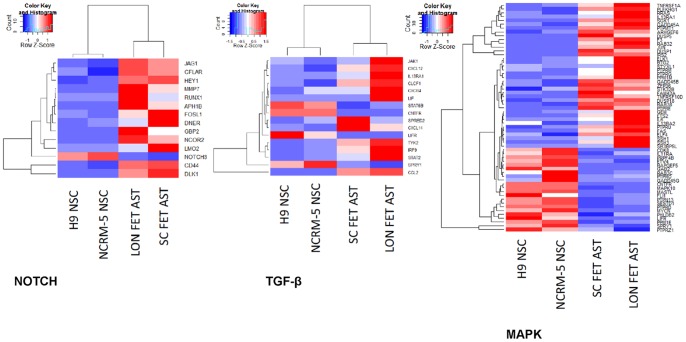
Heatmaps for differentially expressed genes from (A) NOTCH, (B) TGF-beta, and (C) JAK-STAT pathways. Heatmaps are shown for genes differentially expressed between Lonza fetal astrocytes (LON FET AST), Sciencell fetal astrocytes (SC FET AST), H9 NSCs, and NCRM-5 NSCs. All genes show at least 2-fold difference between at least one fetal astrocyte and NSC sample.

For the NOTCH pathway nine genes are upregulated in both fetal astrocyte samples and only one gene is downregulated (Figure 3A). Amongst the upregulated genes HEY1 and the atypical ligands DLK1 and DNER are similarly upregulated in NSC-derived astrocytes. HEY1 seems to be relatively astrocyte specific as it is expressed at low levels in differentiated neurons while DLK1 and DNER show robust expression in neurons. The only NOTCH gene downregulated in fetal astrocytes relative to NSCs is NOTCH3 but it does not show similar regulation in comparisons between NSCs and NSC-derived and astrocytes. Despite the fact that there is more upregulation of selected members of the NOTCH pathway in fetal astrocytes, most of the components of the canonical NOTCH pathway are present NSCs, fetal, and NSC-derived astrocytes and neurons suggesting the pathway is active in all these cells but perhaps utilized in different manners.

#### TGF-beta pathway

A total of five genes in the TGF-beta pathway are upregulated and three downregulated in both fetal astrocyte samples (Figure 3B). None of the genes upregulated in fetal astrocytes is similarly regulated in NSC-derived astrocytes although two of the downregulated genes (BMP7, TGFBR3) are downregulated in NSC-derived astrocytes. TGFBR3 is a negative regulator of the TGF-beta pathway so this along with the other array results suggest this pathway is less active in NSCs [35]. It should be noted the TGF-beta pathway seems to be very active in Lonza fetal astrocytes as eight additional genes in this pathway were upregulated only in this sample. These cells appear capable of signaling through BMP2/4, GDF10, and TGFB2/3 whereas Sciencell astrocytes only signal through TGFB 2/3. NSC-derived astrocytes do not seem to actively signal via any of these ligands except perhaps TGFB1 which is expressed at low levels.

#### JAK/STAT Pathway

Six genes from the JAK/STAT pathway are upregulated in fetal astrocytes and four are downregulated (Figure 3C). Cytokines appear to be upregulated while several growth factor receptors (LIFR, CNTFR) are downregulated in fetal astrocytes. This appears to be unique to the fetal astrocytes as none of these are similarly regulated or in the case of many of the upregulated cytokines even expressed in NSC-derived astrocytes. It is noteworthy that the LIF and CNTF receptors are both upregulated in the NSCs with the latter not even being expressed in fetal astrocytes but only the LIF ligand is expressed in any of these samples and it is found only in Lonza fetal astrocytes. Many of the other signaling and downstream components of the JAK/STAT pathway (JAKs, STATs, IRFs, TYKs) are found in fetal astrocytes and NSC-derived astrocytes and neurons suggesting the pathway is active in these cells as well.

#### MAPK Pathway

**Figure 4:**
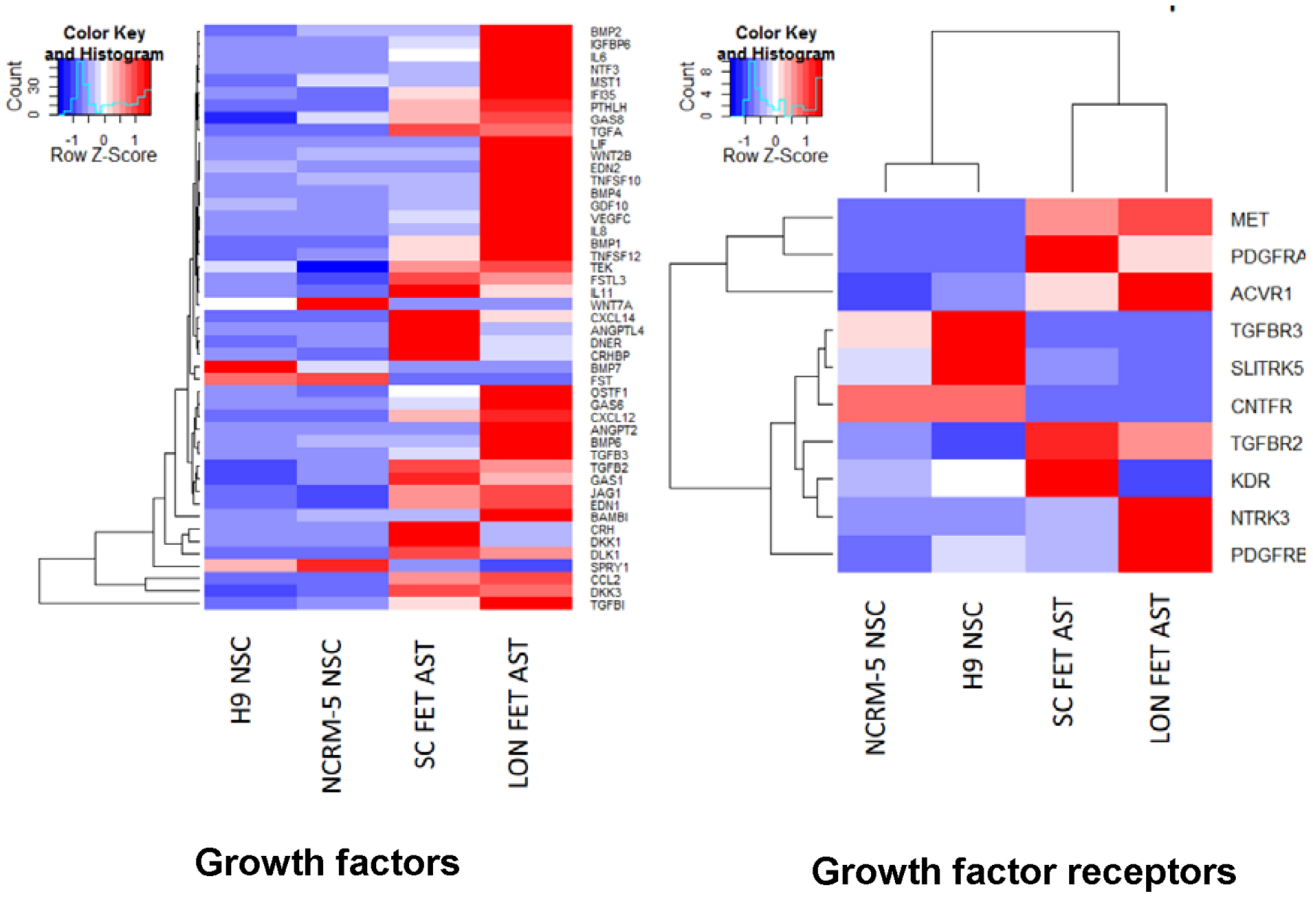
Heatmaps for differentially expressed genes from MAPK pathway. Heatmaps are shown for genes differentially expressed between Lonza fetal astrocytes (LON FET AST), Sciencell fetal astrocytes (SC FET AST), H9 NSCs, and NCRM-5 NSCs. All genes show at least 2-fold difference between at least one fetal astrocyte and NSC sample.

When examining the MAPK pathway a total of 20 genes are upregulated and 15 downregulated in fetal astrocytes (Figure 4). Genes that are upregulated include a receptor tyrosine phosphatase implicated in brain function (PTPRE), IRS2, and the dual specificity phosphatase DUSP18 [36]. GO analysis and the relatively large number of upregulated genes in this pathway suggest that MAPK signaling is critical for maintenance of fetal astrocytes.

#### Growth factors and growth factor receptors

**Figure 5:**
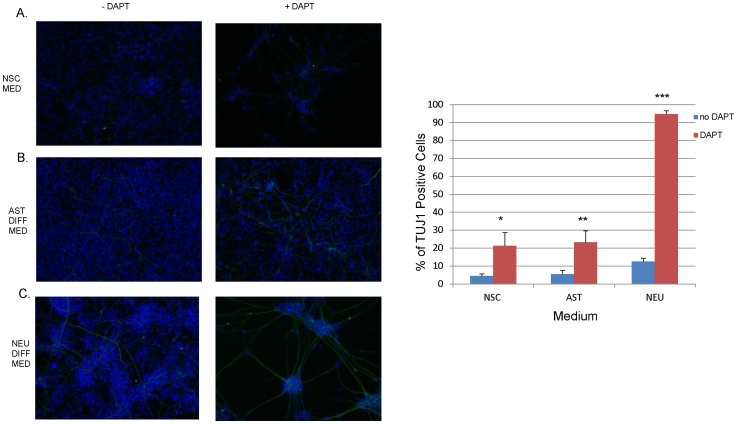
Heatmaps for genes differentially expressed growth factor and growth factor receptor genes. Heatmaps are shown for genes differentially expressed between Lonza fetal astrocytes (LON FET AST), Sciencell fetal astrocytes (SC FET AST), H9 NSCs, and NCRM-5 NSCs. All genes show at least 2-fold difference between at least one fetal astrocyte and NSC sample.

Eighteen growth factors and three growth factor receptors are upregulated in fetal astrocytes whereas only three of each is downregulated in this cell type relative to NSCs (Figure 5). As described above many of the upregulated genes are ligands of the TGF-beta family. Additionally many cytokines are also upregulated in fetal astrocytes. The PDGRA and MET genes are also upregulated in fetal astrocytes which is particularly interesting because of the potential cross-talk between these receptors [37]. It is possible PDGF signaling is important in fetal astrocytes as the PDGF ligand PDGFC and PDGFD are also expressed.

### Transcription factor binding site analysis of transcription factors overexpressed in astrocytes

There were several structural genes that were highly enriched in our dataset. We examined sequences up to 8 kb upstream of the translation start site for five of these genes (GFAP, CD44, LGALS3, DUSP23, and S100A6) to determine what transcription factors bound all of these genes and were also expressed in both fetal astrocyte samples (Table 6). The 24 genes meeting these criteria included E2F2 and TCF7L2 which were identified as being important for gliogenesis in mice [38], and NFIC, SMAD3, and two STAT genes all of which are known to have critical roles in astrocyte development. Additionally, conditional knock-out of mouse serum response factor (SRF) in NSCs resulted in significant loss of astrocytes in the mouse brain [39]. The fact that these genes were identified suggests that some of the other genes in this group may have an important role in astrocyte development and maintenance.

**Table 6:** Transcription factors that are expressed in both fetal astrocyte samples and have binding sites within 8-enriched structural genes.

SYMBOL	LONZA FET AST	SCIENCELL FET AST	NCRM-5 NSC	H9 NSC
ATF1	180	205	151	268
CREB1	1272	1269	1593	1619
E2F2	210	301	1064	834
E2F3	884	1095	1633	1570
EGR1	3970	3779	1197	443
GABPA	130	137	184	121
HBP1	319	177	220	170
LEF1	1122	219	6	222
NFIC	242	175	9	7
NFKB1	1176	684	591	878
RXRA	379	291	464	446
SMAD3	1171	444	687	611
SP1	232	302	387	343
SRF	1655	843	1415	1051
STAT1	703	627	487	353
STAT3	538	369	491	399
TCF3	622	819	1309	1159
TCF7L2	133	182	267	269
TGIF1	221	182	247	258
ZFP161	285	132	371	336
ZNF187	191	161	142	102
ZNF281	457	703	707	924
ZNF410	756	665	775	742

### Inhibition of NOTCH signaling in NSCs

**Figure 6:**
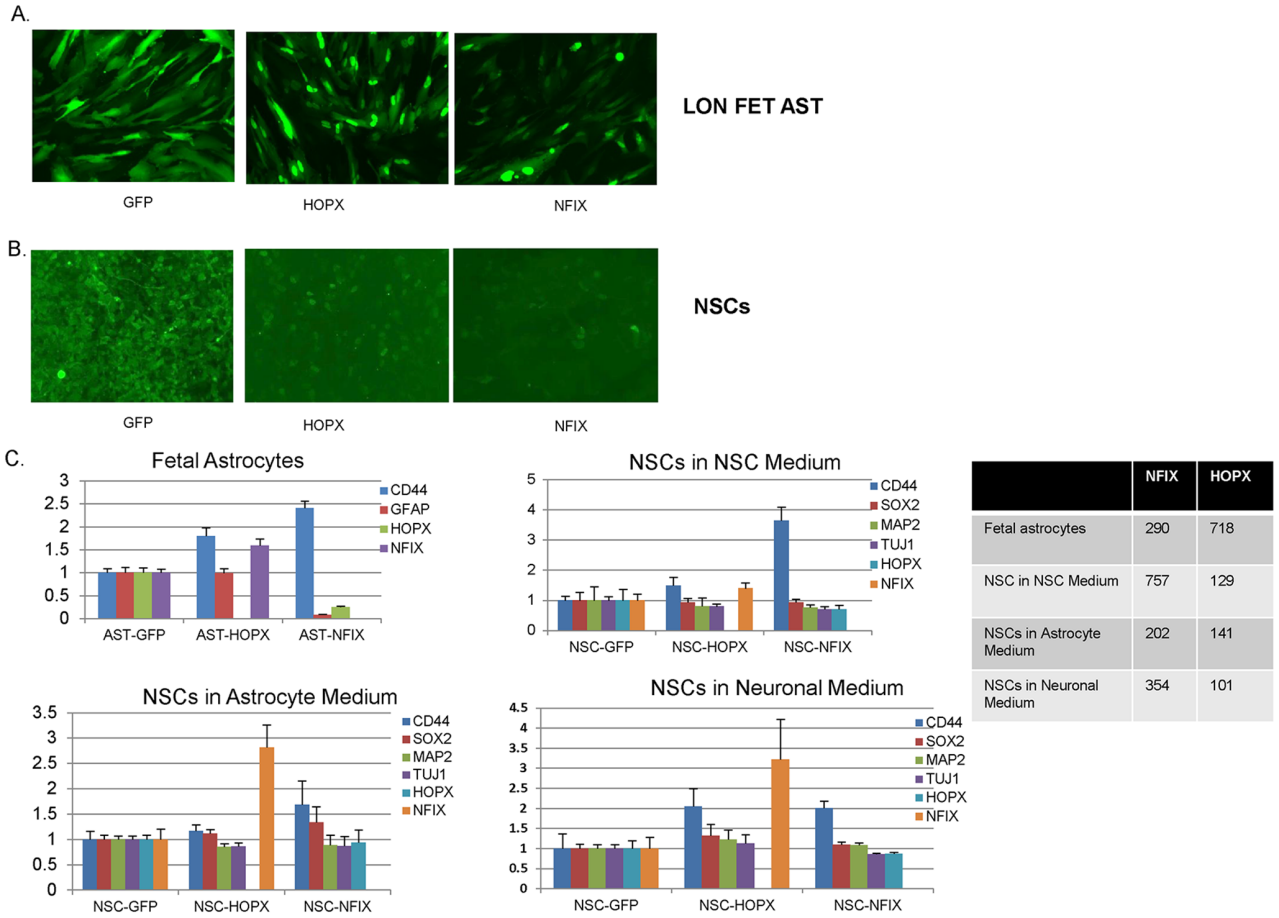
DAPT treatment of NSCs promotes neuronal differentiation. NSCs were stained for TuJ1 (green) after being grown for four days +/− DAPT in, (A) NSC medium (NSC MED), (B) astrocyte differentiation medium (AST DIFF MED), and (C) neuronal differentiation medium (NEU DIFF MED) with an overlay of HOECHST staining (blue) to display all cells, (D) table quantifying the percentage of TuJ1 positive cells for each treatment group. *p-value = 0.03, **p-value = 0.01, ***p-value = 0.0001.

Due to the previous work indicating that NOTCH signaling plays a central role in astrogenesis and our own findings that there is upregulation of certain pathway components in astrocytes relative to NSCs we assessed the effects of inhibiting this pathway. Multiple genes involved in all stages of the NOTCH pathway were found in both in fetal astrocytes and NSCs. In order to better understand the role of Notch signaling in cell fate choice in NSCs we used DAPT, a small molecule inhibitor of the Notch pathway. After four days of culture in NSC, astrocyte, or neuronal growth medium iPSC-derived NSCs exposed to DAPT had increased expression of the neuronal marker β-III-tubulin (TuJ1) compared to untreated cells (Figure 6A–C). No GFAP staining was observed in either the treated or untreated cells and similar results were found in an ESC-derived NSC line. The percentage of TuJ1+ cells was quantified and found to be significantly different across all cell culture medium groups (Figure 6D). The treated cells particularly those in neuronal differentiation medium also displayed enhanced cell death. We also treated fetal astrocytes with DAPT but treatment did not result in the appearance of TuJ1+ cells or increased cell death (data not shown).

### Overexpression of astrocyte specific transcription factors in NSCs

**Figure 7:**
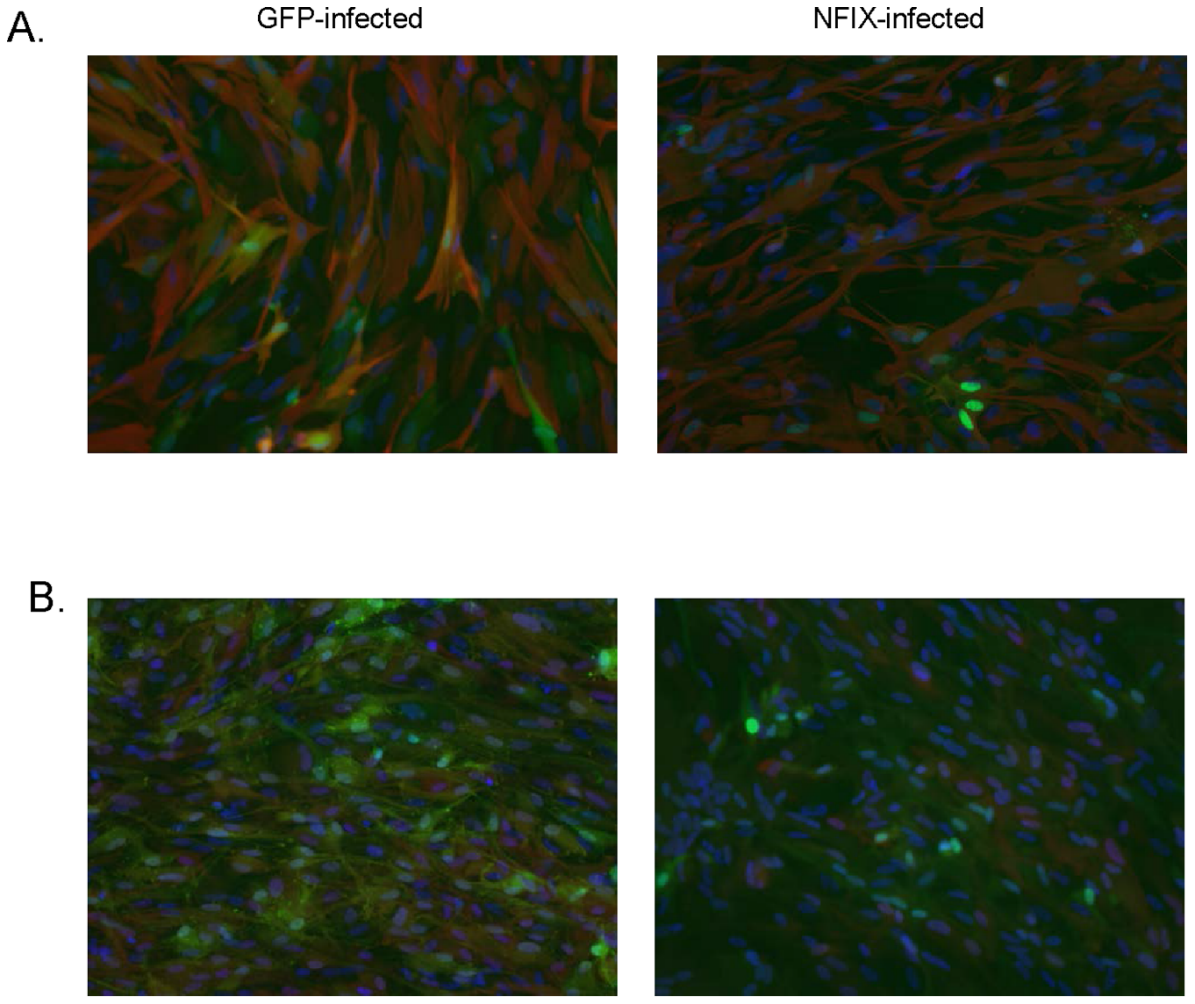
Lentiviral overexpression of NFIX and HOPX in fetal astrocytes and NSCs. GFP expression in GFP, NFIX-nuclear GFP, and HOPX nuclear-GFP cells in (A) Lonza fetal astrocytes (LON FET AST), and (B) NSCs. (C) Tables showing qPCR fold-changes of selected genes in fetal astrocytes and NSCs grown in NSC, astrocyte, or neuronal medium relative to b-actin with GFP lentivirus. The table shows the relative overexpression levels of the NFIX and HOPX lentiviruses.

Our microarray data and previous studies indicate that NFIX and HOPX are critical for initiation of transcription programs that promote astrocyte differentiation and/or maintenance. To better understand the roles of these genes in astrocyte biology we transduced lentivirus from NFIX and HOPX, along with a GFP control, into fetal astrocytes and NSCs and assayed for expression of selected markers by qPCR. NSCs were further split three days post-infection and grown in NSC, astrocyte, and neuronal medium for an additional three days. Fetal astrocytes were readily infected as evidence by GFP expression while NSC infection occurred at lower frequency (Figure 7A–B). However, all culture conditions/cell types had strong overexpression of HOPX and NFIX (see Table with Figure 7C).

Expression of the early astrocyte marker CD44 increased ∼2-fold with both HOPX and NFIX overexpression in fetal astrocytes (assayed three days post-infection). Somewhat surprisingly NFIX overexpression decreased GFAP transcripts ∼90% and HOPX ∼70% in fetal astrocytes. For NSCs we saw a similar modest increase in CD44 transcripts in all culture conditions with NFIX overexpression and a ∼2-fold increase with HOPX in neuronal medium. HOPX also upregulated NFIX expression 2.5–3 fold in astrocyte and neuronal medium.

**Figure 8:**
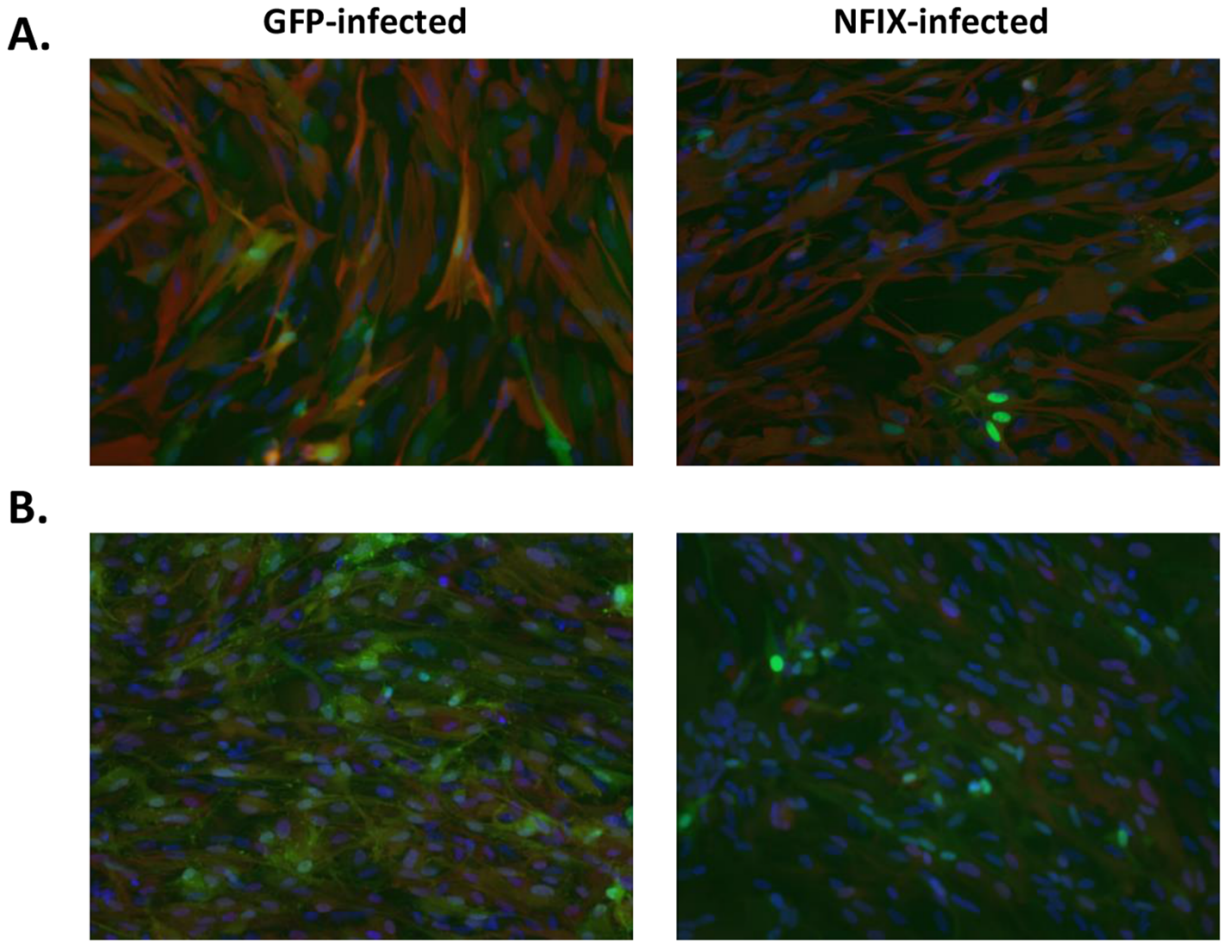
GFAP and HOPX expression in NFIX infected fetal astrocytes. Fetal astrocytes were infected with GFP or NFIX and stained with either (A) GFAP (red), or (B) HOPX (red) antibodies. HOECHST staining (blue) was overplayed to display all cells and GFP (green) to display infected cells.

As we observed such a large decrease in GFAP and HOPX RNA expression in fetal astrocytes we performed immunofluorescence to determine if protein levels also decreased. We were unable to detect any significant differences in GFAP or HOPX staining between GFP and NFIX infections (Figure 8A–B). Upon re-examining the raw qPCR data it appears that the levels of GFAP and HOPX expression in normal (GFP-treated) astrocytes is so high that even a large decrease with NFIX infection still leaves highly abundant levels of RNA for these genes such that protein levels between GFP and NFIX infected groups are not affected.

## Discussion

We have carried out the first study examining gene expression in human fetal astrocytes. Previous studies of astrocyte expression have used murine astrocytes [16], human glial progenitors [17], or astrocytes derived from PSCs [18]. Our goal was not to do a comprehensive survey of genes differentially expressed in astrocytes but identify clear astrocyte specific markers, core pathways and transcription factors important for astrocyte development and maintenance, and compare our results to those of previously published datasets. We reasoned that to achieve this goal for identifying astrocyte markers, each marker should be at least 5-fold enriched relative to NSCs, be expressed in the other dataset of PSC-derived astrocytes, and have very low expression levels in other NSC-derived neuronal and NSC gene expression datasets [18], [20]. Using these criteria, we identified 24 genes that appear to be enriched in astrocytes compared to NSCs and neurons differentiated from NSCs. The fact that known astrocyte markers such as GFAP, CD44, and NFIX are in this list makes us confident that many of the other genes are also astrocyte specific. Other astrocyte enriched genes on the list known to have roles in astrocyte function include LGALS3, CRYAB, S100A6, TGFB3 [40]–[43]. We have previously reported that another gene on this list, HOPX, is enriched in PSC-derived astrocytes [18] and LHX2 and PRRX1 are enriched in NSC-derived and mouse astrocytes [15], [18]. Several Notch pathway genes are on this list including HEY1 and a newly described member of this pathway BEND6. HEY1 has previously been shown to drive generation of astrocytes at later stages of mouse brain development [44] and BEND6 was found to inhibit self-renewal in NSCs from the mouse cortex and promote neurogenesis through antagonism of the Notch signaling pathway. Due to the importance of Notch signaling in astrogenesis BEND6 could be a novel mediator of this pathway as NSCs differentiate to glia [25].

We also identified nearly 100 transcription factors that are differentially expressed between astrocytes and NSCs. Some like HEY1 are known to be effectors of NOTCH signaling and others like the NFI family of transcription factors are known to modulate the NOTCH effector HES5 [10]. Additional transcription factors that could play important roles in gliogenesis include LHX2, PRRX1, and HOPX. Each of these was also found to be present in mouse astrocytes and astrocytes differentiated from NSCs [16], [18]. Interestingly, fetal astrocytes and not NSC-derived astrocytes expressed EMX2, FOXG1, and OTX2 which are not found in NSC-derived astrocytes. These three genes are well known as forebrain markers and as the astrocytes used in this study were from the cortex it is possible that they represent region specific markers for cortical astrocytes [30], [31].

We overexpressed NFIX and HOPX lentivirus in fetal astrocytes and NSCs finding that both increased CD44 expression in both cell types while NFIX greatly diminished GFAP and HOPX expression in fetal astrocytes. However, this decrease of GFAP and HOPX RNA was not seen at the protein level. It appears that there is a huge excess of GFAP and HOPX transcripts produced in fetal astrocytes such that even a large decrease in the transcription of these genes does not affect the final levels of protein that is synthesized. Singh et al. [12] have shown that an NFIX splice variant but not the more common NFIX mRNA we used can upregulate the GFAP promoter in HEK293 cells and primary astrocytes. Further work will be necessary to determine exactly how NFIX regulates HOPX and GFAP but as HOPX did increase NFIX RNA expression in NSCs grown in several culture conditions it is quite possible that NFIX and HOPX are part of a regulatory loop that is important for maintenance of astrocyte identity.

We further studied the role of transcription factors by extracting binding sites for transcription factors for four of our putative fetal astrocyte markers. This search yielded several transcription factors that are expressed in fetal astrocytes including E2F2 and TCFL2 which have been shown to be important for gliogenesis in mice [38], the NFI member NFIC, and SRF another gene that is critical for astrocyte generation.

When examining pathways we found that for the NOTCH pathway two non-canonical ligands, DLK1 and DNER, are highly overexpressed in astrocytes. DNER activates NOTCH signaling in Bergmann glia and has been shown to induce glial and neuronal differentiation in zebrafish while inhibiting NSC proliferation [45]–[46]. Conversely, DLK1 has been shown to inhibit the NOTCH signaling pathway [47]–[48]. To test the role of NOTCH signaling in NSCs we treated them with DAPT and found that in a short period of time it can promote neurogenesis in several different culture conditions. Inhibition of NOTCH by DAPT also increased cell death particularly in neuronal medium. Similar results have been reported when neural rosettes or NSCs cultured are treated with DAPT [49]–[50]. Future studies will need to resolve whether the ability of NOTCH signaling to both stabilize the NSC state and promote astrogenesis is due to differential coupling with HES/HEY genes and involvement of other modulators of Notch signaling such as BEND6.

The microarray analysis of pathways revealed interesting results for the TGF-beta pathway. The TGF-beta is highly active in one subset of fetal astrocytes and relatively inactive in NSCs and NSC derived astrocytes. Thus, activation of this pathway in NSCs may enhance astrocyte differentiation. Similarly modulation of JAK/STAT, MAPK, and PDGF signaling in NSCs is likely to have significant effects on gliogenesis.

In conclusion, we have elucidated gene expression in fetal astrocytes and found that they express many previously described astrocyte marker genes and also express signaling components from pathways known to be active in this cell type. We have also identified potentially new human astrocyte markers that will be of use in distinguishing astrocytes from other neural cells and transcription factors that could be the starting point for identifying the core transcriptional circuitry that defines a human astrocyte. Our results underscore the importance of a group of transcription factors in astrocyte development and maintenance and reinforce the importance of the NOTCH, TGF-beta, JAK/STAT, and MAPK signaling pathways in this cell type. As such this dataset will be an important building block for further understanding human astrocyte biology.

## Supporting Information

Table S1 **List of antibodies used.**
(XLSX)

Table S2 **Primers used for qPCR.**
(XLSX)

Table S3 **Genes up at least 5-fold in pairwise comparisons between two fetal astrocyte and two NSC samples.**
(XLSX)

Table S4 **Expression levels of transcription factors in fetal astrocytes, NSCs, and neurons and astrocytes differentiated from NSCs.**
(XLSX)

Table S5 **Expression levels of chromatin binding genes in fetal astrocytes, NSCs, and neurons and astrocytes differentiated from NSCs.**
(XLSX)

Table S6 **Expression levels of NOTCH signaling pathway genes in fetal astrocytes, NSCs, and neurons and astrocytes differentiated from NSCs.**
(XLSX)

Table S7 **Expression levels of TGF-beta signaling pathway genes in fetal astrocytes, NSCs, and neurons and astrocytes differentiated from NSCs.**
(XLSX)

Table S8 **Expression levels of JAK/STAT signaling pathway genes in fetal astrocytes, NSCs, and neurons and astrocytes differentiated from NSCs.**
(XLSX)

Table S9 **Expression levels of MAPK signaling pathway genes in fetal astrocytes, NSCs, and neurons and astrocytes differentiated from NSCs.**
(XLSX)

Table S10 **Expression levels of growth factors and growth factor receptor genes in fetal astrocytes, NSCs, and neurons and astrocytes differentiated from NSCs.**
(XLSX)
